# Pattern Classification in Kampo Medicine

**DOI:** 10.1155/2014/535146

**Published:** 2014-02-20

**Authors:** S. Yakubo, M. Ito, Y. Ueda, H. Okamoto, Y. Kimura, Y. Amano, T. Togo, H. Adachi, T. Mitsuma, K. Watanabe

**Affiliations:** ^1^Committee for Terminology and Classification, Japan Society for Oriental Medicine, 1-9-18 Kaigan, Minato-ku, Tokya 105-0022, Japan; ^2^Center for Kampo Medicine, Keio University School of Medicine, 35 Shinanomachi, Shinjuku-ku, Tokyo 160-8582, Japan

## Abstract

Pattern classification is very unique in traditional medicine. Kampo medical patterns have transformed over time during Japan's history. In the 17th to 18th centuries, Japanese doctors advocated elimination of the Ming medical theory and followed the basic concepts put forth by Shang Han Lun and Jin Gui Yao Lue in the later Han dynasty (25–220 AD). The physician Todo Yoshimasu (1702–1773) emphasized that an appropriate treatment could be administered if a set of patterns could be identified. This principle is still referred to as “matching of pattern and formula” and is the basic concept underlying Kampo medicine today. In 1868, the Meiji restoration occurred, and the new government changed its policies to follow that of the European countries, adopting only Western medicine. Physicians trained in Western medicine played an important role in the revival of Kampo medicine, modernizing Kampo patterns to avoid confusion with Western biomedical terminology. In order to understand the Japanese version of traditional disorders and patterns, background information on the history of Kampo and its role in the current health care system in Japan is important. In this paper we overviewed the formation of Kampo patterns.

## 1. Introduction

The globalization of health care has not left traditional medicine behind. The World Health Organization (WHO) took the initiative for globalization of traditional medicine by founding the Division of Traditional Medicine in 1972 [1]. In 1978, the Alma-Ata Declaration on Primary Health Care called on countries and governments to include the practice of traditional medicine in their primary health care approach [2]. Thirty years later, traditional medicine is widely available, affordable, and commonly used in many parts of the world.

WHO is presently updating its International Classification of Diseases from the 10th (ICD-10) to 11th edition (ICD-11) [3, 4] and plans to incorporate traditional medicine into this new version. International experts from China, Korea, Japan, Australia, the US, and the EU are involved in this project. The ICD-11 alpha version was released in 2011, and the beta version was released in May 2012, with a version also available on the web [5].

The ICD-11 beta version contains 2 sections on traditional medicine: “traditional disorders” and “patterns” (zheng in Chinese). China and Korea referred to their own national standards to develop these sections. China used the 1995 classification and codes of traditional disorders and patterns of traditional Chinese medicine (GB95) as a national standard. The third edition of the Korean Classification of Diseases of Oriental Medicine (KCDOM3) was incorporated into the Korean modification of ICD-10 (KCD-6) in 2010. KCD-6 was groundbreaking because it was the first publication in which Western biomedicine and traditional medicine shared a common platform in terms of medical statistics.

For Japan's contribution to this edition, the Committee for Terminology and Classification of the Japan Society for Oriental Medicine (JSOM) was responsible for organizing the section on Kampo classification. Kampo covers a wide variety of traditional Japanese medicine including acupuncture and moxibustion, existing before Western medicine was introduced to Japan. In contrast to China and Korea, Japan did not have national standards for reference. To understand the Japanese version of traditional disorders and patterns, background information on the history of Kampo and its role in the current health care system in Japan is important.

## 2. History of Kampo Medicine

Medicines were brought from ancient China to Japan via the Korean peninsula in the 5th or 6th century. While Japanese medicine originally followed the ways of ancient Chinese medicine, Japan adopted Chinese knowledge to suit its own climate and race [6]. Also because not all materials were available, Japan replaced the material to the Japanese herbs and minerals. The first Japanese medical book, “Daidoruijuho,” was a collection of Japanese traditional therapies written in 808.

Further modifications of Japanese traditional medicine occurred during the Edo period (1603–1867) [7, 8]. The medicine of Ming-China was introduced at the beginning of this period and spread widely (Gosei school). During this time, Japanese doctors advocated the elimination of Ming Chinese medicine, instead following the basic concepts of Shang Han Lun and Jin Gui Yao Lue introduced during the later Han dynasty (25–220 AD). The physician Todo Yoshimasu promoted his perspective on these classic texts and rejected the theory developed later in China. His approach emphasized that an appropriate treatment could be administered if a set pattern could be identified, a practice still referred to today as “matching of pattern and formula” (Koho school). Later in the Edo period, another school which integrated both Koho style and Gosei style occurred (Setchu school).

Among these three schools, Koho school influenced most the current Kampo practice in Japan.

In the 18th century, European medicine was introduced in Japan. Modern anatomy was first studied in 1754 by Toyo Yamawaki, a famous Kampo doctor who had acquired an anatomy book from Europe. Toyo Yamawaki respected Yoshimasu, who also knew European medicine. Yoshimasu may have tried to reform Kampo medicine to harmonize it with European medicine.

This trend was followed by other doctors like Seishu Hanaoka (1761–1835), who performed the first surgery with general anesthesia in 1804. This event occurred 42 years before William T. G. Morton successfully performed surgery using ether as a general anesthetic. Hanaoka combined Kampo and European medicines, using Kampo mainly for internal medicine and European medicine for surgery.

The Meiji restoration occurred in 1868, and the new government decided to modernize Japan introducing European culture including medicine. With the passing of the 1874 Medical Care Law, the German model was adopted as the national health care system, and all Kampo-related systematic education was stopped. Kampo practitioners were no longer recognized as official medical professionals; for those interested in becoming physicians, the only option available was to study Western medicine and pass a national examination. Thereafter, the practice of Kampo drastically declined.

After difficult years, physicians like Kyushin Yumoto (1876–1941), Keisetsu Otsuka (1900–1980), and Domei Yakazu (1905–2002) played a key role in reviving Kampo medicine. For Kampo medicine to survive, these physicians had to transform it into a more practical form that the new generation of physicians would also find useful. The modern form of Kampo medicine lost much of its theoretical origin, and emphasis was now being placed on proper prescription of Kampo formulas for treating symptoms. These changes made Kampo conceptually easier to understand for the new generation of physicians trained only with Western medicine. Moreover, the “matching of pattern and formula” methodology made the clinical use of Kampo a more appealing form of treatment.

The result of these efforts was that, by 1967, the first 4 Kampo formulas were approved by the government for coverage under the national insurance system.

## 3. Current Status of Kampo Medicine in Japan

Recent research shows that about 90% of physicians in Japan use Kampo medicines in daily practice, even for cancer patients [9–11]. For women's health, nearly 100% of Japanese obstetrics/gynecology doctors use Kampo medicine [12–14]. Physicians even use Kampo medicine in the university hospital along with high-tech techniques such as organ transplantation or robotic surgery. Physicians often use Kampo medicines along with chemotherapy or radiation therapy for cancer patients. These examples show the magnificent integration of modern Western biomedicine and traditional medicine [15, 16].

Kampo medicine has government-regulated prescription drugs, and now 148 formulas are listed on the Japanese Insurance Program. Kampo practitioners can also use decoctions, selecting several herbs among 243 types covered by the insurance system [17]. In 2001, the Ministry of Education, Culture, Sports, Science and Technology decided to incorporate Kampo medical education into the core curriculum of medical schools. There are 80 medical schools in Japan, all of which now provide Kampo medical education.

## 4. How the “Kampo Medical Classification” Developed Recently in Japan

The Japan Society for Oriental Medicine (JSOM) was founded in 1950 and is the largest academic association for Kampo medicine. The JSOM Committee for Terminology and Classification decided not to use traditional names for disorders in Kampo classification because many of them overlap with Western biomedical terms. Traditional names for disorders are primarily symptoms, such as “headache” or “watery diarrhea.” In contrast, in Western medicine, disease names are based on pathological causes, such as cholera or malaria. Since these diseases have existed for a long time, traditional medicine recognizes these diseases. However, the pathologies of these diseases were unknown when the names were given and so are not reflected in the disease names in traditional medicine. Therefore, it is difficult to map traditional disorder names and biomedical disease names. Sometimes, symptomatic traditional names for disorders are broad and can be mapped to multiple biomedical disease names. Because the restoration of Kampo medicine in Japan was led by physicians, Western biomedical terms were often used instead of the traditional Kampo terms to avoid confusion.

Organ system patterns are very important in medicine in China and Korea. However, Kampo experts in the Meiji (1868–1912), Taisho (1912–1925), and Showa (1926–1989) eras chose not to use organ systems to avoid overlap with biomedical terms. As a result, Kampo medicine is sometimes criticized because of the relative lack of terms to describe patients' conditions. The pathogenesis rather than host reaction is most important in Western biomedicine. In contrast, the host's reaction to the pathogen is the most important factor in traditional medicine. In this regard, Kampo medicine has been developed in harmony with Western biomedicine.

## 5. Kampo Medicine Patterns

Kampo patterns were reconstructed logically according to the ICD principles, which are both jointly exhaustive and mutually exclusive. Several parameters are used for determining Kampo patterns: yin-yang, deficiency-excess, cold-heat, 6 stages of acute febrile diseases, and qi-blood-fluid [18]. Of these, yin-yang, deficiency-excess, cold-heat, and interior-exterior belong to the 8 principles used in Chinese medicine. In China, each component is used in combination with the others to define the pattern, such as “liver yin deficiency pattern,” and is not usually used independently. Among 8 principles, yin-yang is a polysemic word. Sometimes it is used for the sensible temperature in Japan. Under international harmonization, yin-yang is usually a high-level concept of deficiency-excess, cold-heat, and interior-exterior. To avoid confusion, we decided not to use yin-yang for the sensible temperature.

**Figure 1:**
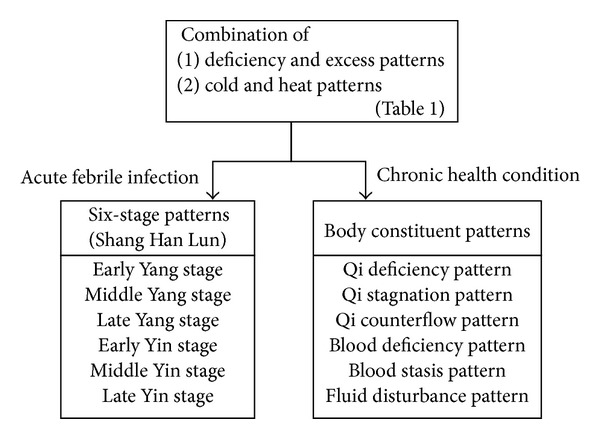
Diagnostic flow used in Kampo medicine. All patients are assigned a specific category as described in Table 1 and then divided into 2 groups according to whether they have acute febrile infectious disease or chronic disease. For acute febrile disease, the 6 stages of Shang Han Lun are very important. For chronic diseases, the host body constituent patterns are very important.

Kampo patterns are determined for all patients according to the flow charts shown in Table 1 and Figure 1. Patient conditions are divided into 2 groups: acute febrile infectious conditions and chronic conditions (Figure 1). A 6-stage pattern, based on Shang Han Lun, is used for describing acute febrile infectious diseases like influenza. Qi-blood-fluid patterns are mainly used for describing chronic diseases.

One issue raised regarding Kampo patterns concerns the “between deficiency and excess” pattern. The deficiency and excess pattern is usually based on the strength of the pathogen. However, in Japan, deficiency and excess patterns are primarily based on the patient's condition. The ancient textbook of Huangdi Neijing (Former Han dynasty; 220 AD to 8 AD) explains that “when the foreign pathogen is strong, it is called as excess, and when body energy is weakened, it is called as deficiency.” The problem with this statement is that deficiency is defined by the strength of foreign pathogens, and deficiency is defined by the energy of the host. Many traditional medical terms are polysemic, mainly due to their long history. However, the deficiency-excess terms are originally polysemic; this has created much confusion.

In Japan, deficiency-excess was originally determined by the strength of the foreign pathogen in the case of acute febrile infectious diseases and by the strength of the body energy in the case of chronic diseases. Additionally, Kampo medicine was used extensively for acute febrile infectious diseases before antibiotics were developed, where the strength of the foreign pathogen was very important. Since the development of antibiotics, Kampo medicine has been used more often for chronic diseases, in which the strength of the body energy is more important. In the modern version of Kampo, the host condition is assigned a high value, while the foreign pathogen is addressed by Western biomedicine. Therefore, the host energy is of greater importance. The need thus arose for the option to designate the body energy level as “neutral” rather than just “deficient” or “excessive.” This issue was raised by Tokaku Wada (1743–1803), a physician in the Edo period [19]. His clinical wisdom was described in “Dosui Sagen” which was published in 1805. In this book, “between deficiency and excess” was described in the type of edema. This idea is thought to have influenced Kazuo Tatsuno (1905–1976) [20, 21] and other physicians in the Showa era. For example, a patient with impaired glucose tolerance appears normal according to the older Kampo designations, even though Kampo medicine is indicated for this condition. In such cases, the “neutral” designation enables acknowledgment of a condition that lies between deficiency and excess.

## 6. Formula Pattern

The formula pattern is also very unique in Kampo medicine. While traditional Chinese medicine (TCM) prescriptions are individualized at the herbal level, Kampo medicine is individualized at the formula level. This practice may have started during the Edo period, as usage of different amounts of herbs was described in a book by Kaibara in 1712 [22]. According to this book, the amount of each herb used in Japan was 1/5 to 1/3 that used in China. Kaibara explained that one of the reasons for this practice was the difficulty in importing herbs from China. Even though alternative herbs available in Japan were used, some had to be imported from China. These differences in the amounts of herbs used are still prevalent. This may explain why Kampo medicine is individualized at the formula level. During the Edo period, doctors carefully studied the roles of formulas and decided the characteristics of each formula. This practice led to Yoshimasu's idea of “matching of pattern and formula.”

Physicians continue to follow this principle today. Clinical trials have been conducted using the same Kampo formula used previously for a specific disease, determining the appropriate Kampo formula based on host patterns. “Matching of pattern and formula” has thus been shown to be a sophisticated approach.

By 1967, the first 4 Kampo formulas were approved by the government for coverage under the national insurance system, and 148 are now listed.

The acceptance of Kampo formulas into the national health insurance system marked the start of the exponential growth of Japan's market in Kampo medicines. Between 1976 and 1992, the sales of Kampo medicine grew more than 10-fold in Japan (Japan Kampo Medicine Manufacturers Association, 2007) [23].

With such a rapid increase in the number of Kampo drug products sold, the government and pharmaceutical industry needed to ensure that high standards were maintained. In 1987, the government established the Good Manufacturing Practice (GMP) law to ensure safety in manufacturing processes, including the production of Kampo formulas. The stringent manufacturing process for Kampo medicine has increased the legitimacy of this modality, as people can now expect uniformity and high quality from the different formulas. This facilitates “matching of pattern and formula,” because if the formulas are not stable, it is very difficult to consistently match pattern to formula.

## 7. Future Challenges

Even though all 80 medical schools in Japan have incorporated Kampo medical courses into their curricula, the number of such courses is very small compared to that of Western biomedicine courses. Postgraduate and continuous Kampo medical education have not been established. Statistics indicate that Kampo formulas are used in daily practice by 90% of physicians, which represents over 260,000 physicians. However, the number of Kampo experts certified by the JSOM is only 2150. This great discrepancy means that most physicians use Kampo formulas based on Western biomedical disease diagnoses without deep consideration of patterns. Further education is necessary for the users of Kampo formulas.

Another concern for the future is the coding rule used for the qi-blood-fluid pattern. Deficiency, excess, and between deficiency and excess are mutually exclusive. Likewise, cold-heat and the 6 stages are mutually exclusive in the same category. However, several abnormalities in qi-blood-fluid may exist in 1 patient. We conducted a small clinical trial without establishing any coding rules. Some doctors provided only 1 code for the qi-blood-fluid pattern, while others provided 4 codes. For more accurate statistics, coding rules should be developed and training in coding should be imparted.

In terms of international comparisons, Kampo patterns are too simple compared to TCM and traditional Korean medicine (TKM). Organ system patterns are particularly lacking in Japan. However, in ICD-11, all the patterns will be presented on the common platform of Western biomedicine. Some organ system patterns can be linked to Western biomedicine disease codes, even though they do not map one-to-one. ICD 11 has terminology that is novel to ICD. This allows ontology software precisely describe the content of each term and links the different codes to each other. The next stage of ICTM development will be field testing. We expect that the international field test will allow for international comparisons.

## 8. Conclusion

Kampo patterns are rather unique compared to Chinese or Korean patterns. There are 2 explanations for this difference. First, Kampo medicine was separated from the theory of the Ming dynasty and then reestablished based on Shang Han Lun theory during the Edo period. Second, Kampo medicine is used in combination with Western biomedicine by licensed doctors in Japan. Kampo terminology was redeveloped in order to avoid confusion with Western biomedicine.

## Figures and Tables

**Table 1:** Combinations of deficiency-excess and cold-heat patterns.

Components	Cold	Heat	Between cold and heat	Tangled cold and heat
Deficiency	Cold, deficiency	Heat, deficiency	Between cold and heat, deficiency	Tangled cold and heat, deficiency

Excess	Cold, excess	Heat, excess	Between cold and heat, excess	Tangled cold and heat, excess

Between deficiency and excess	Cold, between deficiency and excess	Heat, between deficiency and excess	Between cold and heat, between deficiency and excess	Tangled cold and heat, between deficiency and excess

Regardless of acute or chronic health conditions, all patients are classified into 1 of these 12 combinations. Very limited combinations are used for acute diseases.

Between deficiency and excess; neutral in “deficiency and excess”; between cold and heat; neutral in “cold and heat”; tangled cold and heat; mixture “cold and heat,” for example, cold foot and hot flush on face.
